# School food environment interventions for health and sustainability

**DOI:** 10.1002/14651858.CD015882

**Published:** 2026-03-17

**Authors:** Anna Leibinger, Nicole Holliday, Carmen Klinger, Xiao Tan, Laura Busert-Sebela, Lukas Schwingshackl, Eva Rehfuess, Solange Durao, Peter Philipsborn

**Affiliations:** Chair of Public Health and Health Services Research, Institute of Medical Information Processing, Biometry and Epidemiology (IBE), Faculty of MedicineLMU MunichMunichGermany; Pettenkofer School of Public HealthMunichGermany; Professorship of Public Health and Prevention, TUM School of Medicine and HealthTechnical University of MunichMunichGermany; Chair of Public Health Nutrition, Faculty of Life SciencesUniversity of BayreuthBayreuthGermany; Institute for Evidence in Medicine, Medical Center—University of Freiburg, Faculty of MedicineUniversity of Freiburg, Freiburg, GermanyFreiburgGermany; Health Systems Research UnitSouth African Medical Research CouncilCape TownSouth Africa

## Abstract

This is a protocol for a Cochrane Review (intervention). The objectives are as follows:

**Primary objective:**

To assess the effects of interventions that change the school food environment on health, nutritional, and educational outcomes in children aged 2 to 19 years, as well as on sustainability outcomes (food waste and greenhouse gas emissions).

**Secondary objective:**

To examine how the effectiveness of school food environment interventions varies by schooling level, income level of the country, presence of co‐interventions, socioeconomic status, sex/gender, and rurality.

## Background

### Description of the condition

Healthy diets are a key determinant of human health and well‐being. They play a critical role in preventing malnutrition in all its forms; reduce the risk of non‐communicable diseases (NCDs), including cardiovascular disease, type 2 diabetes, and diet‐related cancers; and can have a positive impact on cognition and emotion [1, 2, 3]. Beyond health, policies that promote healthy dietary patterns can also have a positive impact on the sustainability of the food system [4].

To support healthy diets, many national and international bodies have developed food‐based dietary guidelines that provide evidence‐informed recommendations for optimal dietary intake. These generally recommend high intakes of fruits, vegetables, whole grains, legumes, and nuts; moderate consumption of dairy and other animal‐based foods such as white meat and fish; and low to no intake of free sugars, saturated fats, red and processed meats, as well as energy‐dense and nutrient‐poor processed foods such as sugar‐sweetened beverages, confectionary, and refined grains [5, 6]. Increasingly, these guidelines also incorporate aspects of environmental sustainability [4, 7].

Population‐level dietary intake diverges markedly from these dietary recommendations across all regions worldwide. Global trends indicate low consumption of recommended foods and higher than recommended intake of less healthy foods and nutrients [1]. This contributes to a rising burden of chronic diet‐related disease [8]. According to the Global Burden of Disease Study 2021, an estimated 2100 million adults (aged 25 or older) were living with overweight or obesity in 2021, with rising rates in all nations worldwide between 1990 and 2021 [8]. An estimated 508 million individuals were living with type 2 diabetes in 2021 [9]. Around 18% of individuals aged 5 to 14 years and 20% of those aged 15 to 24 years globally were estimated to be living with overweight or obesity in 2021, totaling approximately 493 million children, adolescents, and young adults [10]. This reflects a doubling in prevalence since 1990. Over the same period, the estimates for the prevalence of obesity alone more than tripled, rising from 2% in 1990 to nearly 7% in 2021 for these age groups combined [10]. Overall, suboptimal diets are a leading cause of global morbidity and mortality [1, 11].

One key driver of the current global burden of diet‐related disease is the so‐called nutrition transition—a shift from traditional diets toward those high in processed foods, refined grains, added sugars, and animal‐based products. This transition has already occurred to a large extent in high‐income countries and is now advancing rapidly in low‐ and middle‐income countries (LMICs) [12]. Particularly in LMICs, this shift has contributed to an increasing prevalence of overweight, obesity, and other NCDs. Undernutrition has decreased markedly over several decades, but has more recently shown a slowing decline in many world regions, and persists at high levels [12]. Recent evidence indicates that short‐term food insecurity is widespread among school‐aged children and adolescents. Analysis of school‐based survey data from 95 countries shows that approximately one‐quarter of students aged 11 to 14 years and one‐third of students aged 15 to 18 years reported food insecurity in the previous month, and that these experiences were consistently linked with poorer diet quality, more missed school days, less physical activity, and adverse mental and behavioral outcomes [13].

These simultaneous trends—rising rates of overweight and obesity alongside persistent undernutrition and widespread short‐term food insecurity—mean that many populations are now experiencing a double burden of malnutrition—the coexistence of both undernutrition and overnutrition within the same communities, households, and individuals over the life course [12, 14]. Unless mitigated, the continuation of the nutrition transition is likely to drive further increases in the burden of chronic disease in the future [15].

In parallel, the current global food system is placing unsustainable pressure on planetary resources. Food production and consumption are major drivers of biodiversity loss, freshwater use, soil degradation, and greenhouse gas emissions [16, 17, 18]. It is estimated that the food system is responsible for approximately 30% of global greenhouse gas emissions and 70% of freshwater withdrawals [17, 19]. Diets high in red and processed meats are particularly associated with negative environmental impacts and have also been linked to adverse health outcomes [4, 20, 21].

### Description of the intervention and how it might work

A shift towards healthier and more sustainable diets can help address the public health challenges described above. International organizations, including the World Health Organization (WHO) and the Food and Agriculture Organization (FAO), recommend a variety of policies and interventions to support healthier and more sustainable diets. Among these, school‐based interventions have been deemed particularly important due to their potential to reach large numbers of children during a formative period of life [22, 23, 24].

Schools are an important setting for ensuring sufficient and healthy dietary intake, as students often consume a substantial proportion of their daily energy and nutrient needs in or around school. In 2022, it was estimated that 41% of all schoolchildren in primary schools globally received free or subsidized school meals (18% in low‐income countries, 39% in lower‐middle‐income countries, 48% in upper‐middle‐income countries, and 61% in high‐income countries) [25]. According to a global report of national school food guidelines, many countries set energy standards for school lunches at around 30% of daily requirements, and between 20% and 25% for breakfast and snacks, so that more than 50% of daily calories would be consumed in schools [22].

Globally, many of these programs have primarily been introduced to address child undernutrition and improve educational outcomes, as previous research has found that school meal programs are associated with improved height and weight development in children; increased school attendance, especially among girls in LMICs; increased ability for students to concentrate during the school day; and improved learning outcomes and academic performance [25, 26, 27, 28, 29]. Furthermore, they present an opportunity to support children's long‐term health. Dietary habits established in youth often carry into adulthood; establishing healthy dietary habits in this critical developmental period can therefore be considered a primary prevention strategy for several NCDs [4, 30].

Given the global scale of school nutrition, school food environment interventions are also being increasingly recognized not only as a means to promote healthy eating habits for individuals, but also as an opportunity to advance sustainability and planetary health, particularly if they promote the consumption of environment‐friendly foods [31, 32, 33].

Dietary patterns are influenced by a range of interacting factors, ranging from individual to environmental level, some of which are modifiable [34]. Among these modifiable factors, food environments are considered particularly influential and offer opportunities for intervention to promote healthier and more sustainable eating behaviors [35]. For schoolchildren, this includes the school food environment. We define the school food environment as all the foods and drinks available and accessible to children in and around schools (including offerings by cafeterias, vending machines, snack bars, and nearby vendors) along with the nutritional quality of these foods and any related information or characteristics, such as accessibility, affordability, advertising, and presentation, that might influence choices [36].

Durão and colleagues proposed a number of interventions to improve the school food environment in a WHO‐commissioned systematic review. They examined the effects of these interventions on children’s health and non‐health outcomes [37]. This review is an update and extension of the original non‐Cochrane systematic review by Durão and colleagues, and we will examine the same set of interventions, extending the focus on healthy diets to include sustainability aspects [37]. In this protocol, we will refer to these interventions by their short names, shown in bold in the following list.

1. **Nutrition standards** or rules that determine the quality of food served or sold in and around schools
2. **Marketing restrictions** of unhealthy and unsustainable foods in and around schools
3. **Nudging interventions** promoting healthy and sustainable food behavior in the school environment (e.g. product placement)
4. **Pricing policies** to promote healthier and more sustainable alternatives (e.g. subsidies of healthy foods; higher cost of unhealthy options)
5. **Direct food provision** to students in schools (e.g. meal programs; vegetable and fruit distribution)

These interventions share several common mechanisms, as they change one or more aspects of the school food environment. They function within the context of the broader food system, impacting how children acquire and consume food. The key areas of influence include food accessibility, affordability, convenience, desirability (or attractiveness), availability, salience, and vendor and product properties [35, 38]. Further details on each of these five intervention types are provided in the following sections, and an overview of how these interventions may contribute to the desired outcomes is presented in a system‐based logic model (Figure 1). This model is based on the logic model proposed by a review of contextual factors influencing the implementation of school food and nutrition policies published by the WHO in 2021 and the system‐based logic model template developed by Rohwer and colleagues [39, 40]. Key definitions of terms used throughout this protocol are provided in Table 1.

**1:**
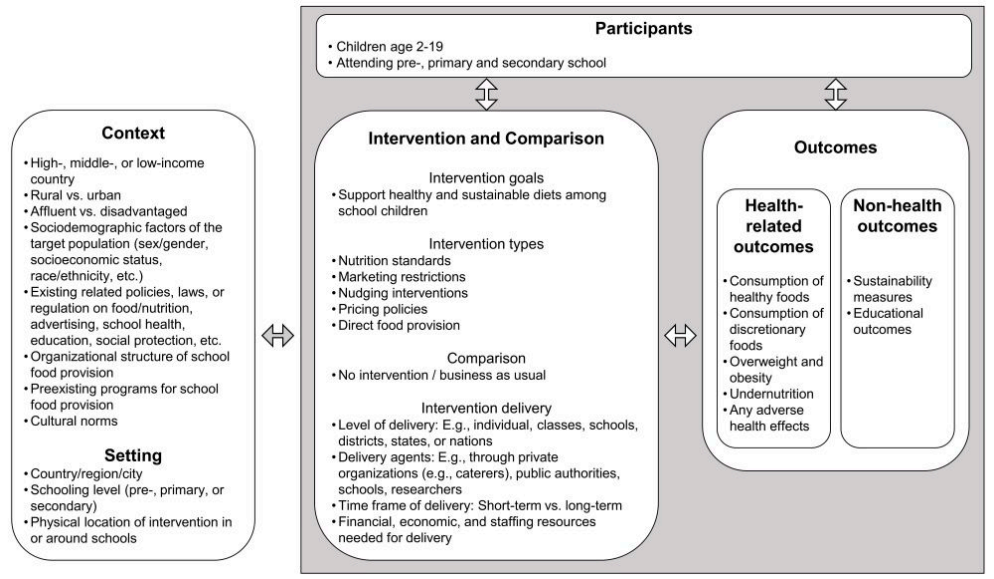
Own creation, based on a logic model by the WHO and a template by Rohwer and colleagues [39, 40].

**1:** Key definitions used in this protocol

Term	Definition
Foods	For the purposes of this review, we define foods as solid foods and non‐alcoholic beverages. This review considers foods provided regularly by the school (e.g. through school meal programs, including those which are provided to students free‐of‐charge, subsidized, and those paid for out‐of‐pocket) as well as competitive foods (i.e. foods available for purchase by students but which are not included as part of the regular school meal program, such as from a vending machine or foods offered around schools) [86].
Discretionary foods	Energy‐dense foods that are high in sugar, sodium, total fats, or trans fats that are not necessary for a healthy diet (e.g. sugar‐sweetened beverages, confectionaries, or chips).
Intervention	For the purposes of this review, we define intervention as any measure altering the school food environment. This includes setting‐based measures (e.g. a decision by an individual school to remove sugar‐sweetened beverage vending machines from its premises) as well as higher‐level policies (e.g. national legislation on school food programs).
Schools	We define schools as including pre‐, primary, and secondary schools. We define primary and secondary schools as educational institutions that typically serve students aged 5 to 18 years, including boarding schools [87]. This includes educational institutions with synonymous names (e.g. basic and elementary for primary, and middle and high school for secondary schools) [88]. We define preschools as center‐based childcare services or pre‐primary education centers which can be publicly or privately managed and are typically run in a purpose‐built facility, have set hours of operation, and are run by trained staff. These exclude purely family‐based arrangements that are not part of a formalized pre‐primary education program [89]. In our definition of schools, we do not include daycares or adult education institutions, such as vocational or technical schools or universities.
Children	For the purposes of this review, we refer to children and adolescents aged 2 to 19 as children, unless explicitly stated otherwise.
Food system	We adhere to the definition of food system by the High Level Panel of Experts for Food Security and Nutrition (HLPE). According to their definition, “a food system gathers all the elements (environment, people, inputs, processes, infrastructures, institutions, etc.) and activities that relate to the production, processing, distribution, preparation and consumption of food, and the outputs of these activities, including socio‐economic and environmental outcomes” [90].
School food environment	We define the school food environment as all the foods and drinks available and accessible to children in and around schools, including offerings by cafeterias, vending machines, snack bars, and nearby vendors, along with the nutritional quality of these foods and any related information or characteristics, such as advertising and presentation or pricing that might influence choices [36].
Low‐ and middle‐income countries (LMICs)	For the purposes of this review, we will use the country income classifications of the World Bank to classify countries by income. We therefore define LMICs as all countries categorized as low‐income, lower‐middle‐income, and upper‐middle‐income, in total encompassing 131 countries in their classification for the fiscal year of 2025 [75].

#### Nutrition standards

According to the FAO, school nutrition standards are “rules, principles and recommendations […] designed to improve the nutritional quality and quantity and/or adequacy of foods and meals available/provided in schools” [22]. Nutrition standards can be nutrient‐based or food‐based. Nutrient‐based standards specify minimum and/or maximum quantities or ranges of energy and nutrients that an average meal or snack should provide, typically based on estimated individual dietary needs. Food‐based standards define specific quantities, portions, or ranges of foods or food groups that should be included or excluded in a meal or snack [22]. Nutrition standards restructure the food environment by changing the product properties, ideally making healthier and/or more sustainable foods more available and accessible to students [41].

#### Marketing restrictions

Marketing of unhealthy foods is delivered through a variety of media, including television, digital platforms, packaging, and in‐store promotions, and includes marketing in or near schools [42, 43]. Marketing restrictions on unhealthy foods to children in and around schools typically involve defining what constitutes unhealthy foods—such as those high in sugar, salt, and fats—and then implementing regulations to limit or ban the marketing of these products in school environments. This can include restrictions on advertising within schools or near schools, during school‐sponsored events, or through the design and packaging of products (e.g. using cartoon characters or other child‐friendly visuals) that are available in or around schools [43]. Marketing techniques, such as advertisements, product placement, and branding, target mostly desirability, associating unhealthy foods with, for example, positive emotions or social status. By removing these cues from school environments, marketing restrictions limit children’s exposure to such marketing and may reduce the desirability of unhealthy products [43, 44].

#### Nudging interventions

Nudging works through indirect suggestions and positive reinforcement that are used to influence the decisions and behaviors of individuals without restricting their freedom of choice or altering the price, for example by placing fruits at eye level to encourage healthier choices [45]. Nudging alters the choice architecture, which means that it structures the food environment in a way that influences how behavioral options are presented, making desired behaviors more noticeable and easier to adopt [46]. Examples of nudging within the school food environment include the placement of certain foods or food groups, or making students or parents, or both, pre‐commit to a food choice [45, 46]. Nudging interventions thus aim to guide students toward healthier and more sustainable food choices by making these more accessible, salient, convenient, and desirable [47, 48].

#### Pricing policies

Pricing interventions aim to promote healthier dietary choices by altering the relative costs of healthy and less healthy foods, such as subsidizing healthy options or increasing the price of less healthy items, or both. Unlike school meal programs that provide free or reduced‐price meals through direct food provision, these interventions focus on the relative affordability of different food groups to shift consumption patterns toward healthier options without eliminating choice. Research suggests that a decrease in price may have a greater impact than a comparable increase in price [49]. Combined interventions that lower the price of healthy foods and increase prices of unhealthy foods may be particularly effective [50].

#### Direct food provision

Direct food provision interventions, such as school meal programs, fruit and vegetable distribution, or the installation of water fountains, aim to improve students’ access to foods and beverages at school. Direct food provision is often available at no or reduced cost to students, benefiting children from families living with low socioeconomic status in particular [25]. These interventions can impact multiple aspects of the food environment by improving accessibility, affordability, and convenience.

### Why it is important to do this review

Improving school food environments has gained increasing attention as a public health and sustainability priority. Global organizations such as the WHO, World Food Programme (WFP), FAO, and the School Meals Coalition have called for stronger evidence and action in this area [22, 23, 25, 51]. In its 2022 *State of School Feeding Worldwide* report, the WFP identified school meal programs as “the world’s most extensive safety net” and highlighted their potential to contribute to healthy and sustainable diets [25]. The FAO’s 2019 report on *Nutrition Guidelines and Standards for School Meals* in 33 LMICs recognizes school meal programs not only as a means to combat malnutrition in all its forms but also as entry points to broader social and educational goals, such as improved learning outcomes [22].

The original systematic review by Durão and colleagues provided an important foundation by synthesizing the available evidence on the effects of school food environment interventions on children’s health and non‐health outcomes [37]. However, given the growing interest and new evidence in this area, an update and extension of this previous work is warranted. In particular, the literature search of the previous review extended only through May 2020, and a preliminary search suggests that several relevant studies have since been published. Additionally, there is growing recognition of the importance of evaluating potential adverse effects and environmental sustainability outcomes, which were not systematically assessed by the original review [25].

By extending the scope to include sustainability‐related outcomes and adverse effects, and by integrating new evidence, this review aims to provide an updated and extended synthesis to inform policy and practice. In addition, this review will enhance the original review by fully applying Cochrane methods and standards (e.g. by conducting all screening, data extraction, and risk of bias assessments in duplicate, and by using the most current tools for risk of bias assessment [RoB 2 and ROBINS‐I V2]). The findings of this review are intended to support decision‐makers and stakeholders in governments, international agencies, non‐governmental organizations (NGOs), and school systems seeking to implement effective and sustainable school food policies.

## Objectives

### Primary objective

To assess the effects of interventions that change the school food environment on health, nutritional, and educational outcomes in children aged 2 to 19 years, as well as on sustainability outcomes (food waste and greenhouse gas emissions).

### Secondary objective

To examine how the effectiveness of school food environment interventions varies by schooling level, income level of the country, presence of co‐interventions, socioeconomic status, sex/gender, and rurality.

## Methods

This review is an update and extension of the non‐Cochrane systematic review by Durão and colleagues on the “Effects of policies or interventions that influence the school food environment on children’s health and non‐health outcomes” [37]. The present review will fully apply Cochrane methods to assess the effects of such interventions on a broader set of outcomes. In addition to updating the evidence base, this review will include potential adverse effects and sustainability‐related outcomes, which were not systematically addressed in the original review.

### Criteria for considering studies for this review

#### Types of studies

We will include studies that quantitatively evaluate the effects of school food environment interventions using experimental and observational designs. Given the challenges of implementing and evaluating public health interventions in school settings—and that we anticipate a limited number of randomized controlled trials (RCTs)—we will apply the following eligibility criteria that are also depicted as a decision tree in Figure 2.

**2:**
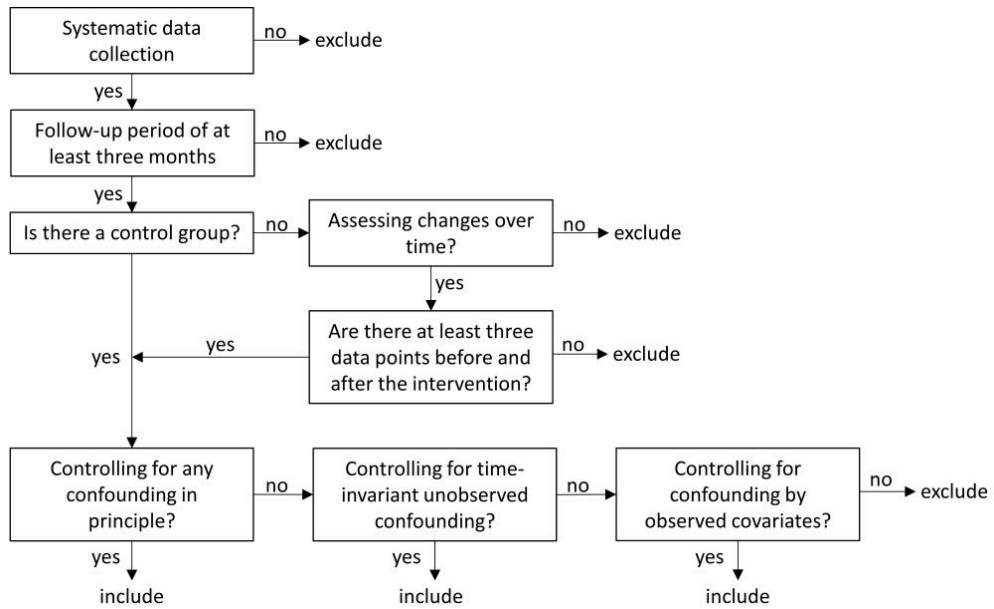
Own creation, based on Reeves and colleagues [66].

1. **Systematic data collection and outcome assessment.** Studies must be based on systematically collected quantitative data on at least one outcome of interest that was measured after the implementation of the intervention.
2. **A follow‐up period and intervention length of at least three months.** We will exclude studies in which the period between the start of the intervention and the final outcome measurement is shorter than three months, as effects of environmental interventions may diminish over time when study participants get used to the new conditions, which may limit long‐term public health impacts. Accordingly, we will exclude studies where the length of the intervention is shorter than three months.
3. **Estimable intervention effects.** The study design must allow the effect of the intervention to be estimated by either i) assessing changes over time (e.g. interrupted time series) in the same or different individuals; in this case, studies will only be eligible for inclusion if there are at least three data points in both the pre‐intervention and post‐intervention periods; or ii) comparing groups or clusters receiving the school nutrition intervention to those that do not (e.g. RCTs, cross‐over RCTs, quasi‐RCTs [where individuals or clusters are assigned to intervention or control groups using a non‐random but systematic allocation method such as alternation, date of birth, school class], controlled before‐and‐after studies, or controlled interrupted time series studies).
4. **Study design classification and control for confounding.** Studies will be classified based on their design features following the approach outlined by Reeves and colleagues that distinguishes studies by how they control for confounding [52]. Studies will be eligible for inclusion if they control for i) any confounding in principle (e.g. RCTs, or quasi‐experimental designs such as regression discontinuity or instrumental variable analyses); ii) time‐invariant unobserved confounding in principle (e.g. difference‐in‐difference or controlled before‐and‐after study designs); or iii) confounding only by observed covariates (e.g. prospective or retrospective cohort studies). We will apply the extended checklist approach from Reeves and colleagues to clarify ambiguities in study design labels that are given by study authors and to assess the intrinsic strength of the causal inference [52].

We will not include systematic reviews, but will consider them for forward and backward citation searches. We will not include mathematical modeling studies.

#### Types of participants

This review will include studies examining children and adolescents aged 2 to 19 years, attending public or private pre‐, primary, or secondary schools in any country. We will include studies that also include children younger than 2 years or young adults older than 19 years if more than 80% of the participants in the study sample are within the age range of 2 to 19 years, or if the study reports data for our age group of interest separately.

If the age of study participants cannot be determined from the included reports, we will contact the study authors. If we do not receive a response, we will list the study as awaiting classification.

We selected the age range of 2 to 19 years as this includes ages when children attend a preschool institution through the end of most compulsory education systems (which typically require children to begin attending school at age 5 and finish secondary school at age 16 to 18). The ages of 2 years and 19 years were included as a buffer to be inclusive of students who may begin preschool/school early or end school later.

#### Types of interventions

We will include studies that evaluate interventions that influence the school food environment. These interventions are listed below.

1. **Nutrition standards.** We will include nutrient‐ and food‐based nutrition standards. The quality of food can be defined along various dimensions, such as healthiness, diversity, portion sizes, or sustainability. School nutrition standards is the term most frequently used for this concept, but there are similar terms to denote the same idea, including school food policies, school meal guidelines, or school nutrition guidelines. For the purposes of this review, these terms will be treated synonymously if the policies they stand for fulfill the aforementioned purpose.
2. **Marketing restrictions.** We will only include interventions that target the school food environment specifically and that restrict marketing of unhealthy or unsustainable foods, or both, in and around schools through any channel, including banners/billboards and sponsoring.
3. **Nudging interventions.** For this intervention type, we will consider any intervention that can be categorized as nudging according to the TIPPME (typology of interventions in proximal physical micro‐environments) tool suggested by Hollands and colleagues and that is not already included in any of the other four intervention types [53]. This includes interventions that i) change the placement of products or objects through changing availability or position; and ii) change the properties of products or objects through altering their functionality, their presentation (visual, tactile, auditory, or olfactory), their size, or the information given about them.
4. **Pricing policies.** We will include any price‐based interventions that change the relative price of more desirable foods (e.g. healthier, more nutritious, more sustainable) and less desirable foods, if they are sold through the same channel (e.g. healthy food subsidies). If healthy meals or snacks are offered through the cafeteria at no cost or at a reduced price, but unhealthy options are still available through a vending machine, or another vendor, then this would not be categorized as a pricing policy, but as direct food provision.
5. **Direct food provision.** We will include any intervention through which schools or other organizations directly provide or distribute food to students in schools, either free of charge, at reduced prices, or at full cost. We anticipate that there will be studies that reduce the price of all meals, or make an already existing school meal program more affordable or free of charge. We will categorize these kinds of interventions as direct food provision and not as pricing policy.

We will include only studies in which one of the five interventions of interest is the clear focus of the study, with decisions made on a case‐by‐case basis. Studies may include complementary multicomponent intervention packages—such as nutrition education, school gardening, deworming or hygiene programs, health status monitoring, or distribution of micronutrient supplements—but will only be included if a primary emphasis is placed on one of the five interventions of interest [22].

We will include studies that compare the intervention of interest with no intervention or business as usual.

### Outcome measures

We will consider studies that report outcome measures within any of the following five critical or two important outcome domains. This list is based on a ranking of outcomes by the WHO guideline development group that we modified and condensed for the purposes of this review (e.g. to include sustainability measures and adverse effects).

For all outcome measures related to food intake, we will consider both self‐reported and objective proxy measures of food intake (e.g. sales and purchasing data), and food intake in school, out of school, and overall. We use the term food to denote both solid foods and non‐alcoholic beverages.

For each included study, we will choose at most two outcome measures per outcome domain, as described in Data extraction and management.

If outcomes are reported at multiple time points, we will use the longest available follow‐up within the intervention period (i.e. the period during which participants are exposed to the intervention). If reported outcomes were only observed after exposure to the intervention has ended, we will use the first time point after exposure to the intervention has ended that is reported. If only a mean treatment effect over the whole or part of the (post‐)intervention period is reported, we will use this mean.

We will use the definitions of healthy (or recommended) and discretionary (or less healthy/unhealthy/discouraged) foods as provided by each individual study. When a study’s classification is clearly inconsistent with current WHO dietary guidelines, we will review it on a case‐by‐case basis and may consider reclassifying the foods accordingly [5].

#### Critical outcomes

1. Consumption of healthy foods (e.g. proportion/quantity/frequency of consumption of healthy foods or meals in general, or specific foods such as fruits and vegetables, nuts, legumes, whole grains)
2. Consumption of discretionary foods (e.g. proportion/quantity/frequency of discretionary foods in general, or of specific foods such as sugar‐sweetened beverages or energy‐dense snacks and sweets)
3. Overweight and obesity (i.e. prevalence or incidence of overweight and/or obesity, changes in body mass index [BMI])
4. Undernutrition (i.e. prevalence or incidence of underweight, stunting, wasting, or micronutrient deficiencies)
5. Any adverse health effects (e.g. eating disorders, body image disorders)

#### Important outcomes

1. Sustainability measures (amount of food waste and greenhouse gas emissions from foods)
2. Educational outcomes (e.g. school absenteeism and educational attainment)

### Search methods for identification of studies

We will conduct searches in English but aim not to exclude any studies based on language, with the current team being able to assess papers published in English, German, French, Italian, Chinese, and Spanish. For publications not written in any of these languages, we will explore translation options.

#### Electronic searches

In addition to databases that contain peer‐reviewed journal articles, we will also search grey literature databases and online trial registries to identify unpublished, ongoing, or other potentially eligible grey literature. We will conduct searches of the following electronic databases.

- Cochrane Central Register of Controlled Trials (CENTRAL)
- MEDLINE via Ovid
- Embase via Ovid
- Web of Science Core Collection
- Scopus
- ERIC via EBSCOhost
- ClinicalTrials.gov
- WHO International Clinical Trials Registry Platform (ICTRP)
- Cochrane Database of Systematic Reviews (CDSR)
- Epistemonikos
- OpenGrey

We will use the search strategy by Durão and colleagues, which we have permission to use [37]. This search strategy was originally developed for PubMed, and we adapted it to MEDLINE/Ovid (see Supplementary material 1). We use the sensitivity‐maximizing version (2023 revision) of the Cochrane Highly Sensitive Search Strategy for identifying randomized trials in MEDLINE (Ovid format) as described in Chapter 4 of the *Cochrane Handbook for Systematic Reviews of Interventions* and extend this strategy with additional search terms to capture other study designs relevant to this review [54]. The extended study design filter used was originally developed for the Cochrane review 'Community‐level interventions for improving access to food in low‐ and middle‐income countries' by Durão and colleagues [55].

For the databases also used by Durão and colleagues in the original review, we will use their adapted search strategies. CDSR, Embase, and ICTRP were the only databases not used by Durão and colleagues, and we will therefore adapt their search strategy for these databases.

In the original review by Durão and colleagues, all databases were searched without a date restriction, and the searches were conducted between 23 April 2020 and 13 May 2020. For each database, we will restrict the search to the timeframe since the date Durão and colleagues searched the same database [37]. This means that we will restrict the search to start on 23 April 2020 for MEDLINE; 24 April 2020 for ERIC; 26 April 2020 for OpenGrey and CENTRAL; 27 April 2020 for Scopus; 27 April 2020 for Web of Science Core Collection; 8 May 2020 for Epistemonikos; and 13 May 2020 for ClinicaTrials.gov. We will not restrict the date of the search for CDSR, Embase, and ICTRP.

#### Searching other resources

We will contact study authors of included studies to ask if they are aware of related ongoing or completed published and unpublished studies.

We will conduct backward and forward citation searches of all included studies. We will search for retractions, errata, corrigenda, and expressions of concern through MEDLINE, Embase, and the Retraction Watch Database (retractiondatabase.org). These searches will be conducted during the review process and repeated close to publication of the review. We will address any post‐publication amendments that we find through forward citation searches or through these targeted searches as follows: a retraction of a study will lead to the exclusion of the study from this review; errata and corrigenda will be used alongside the initially published results (i.e. we will use the corrected version of the study); expressions of concern will be taken into consideration, and we will decide if we will include the respective study on a case‐by‐case basis.

In addition, we will examine previously conducted systematic reviews that include at least one of the five interventions of interest and use these for backward and forward citation searches. For this purpose, we will search the CDSR as well as Epistemonikos, as mentioned above. We will use search strategies by Durão and colleagues for this purpose, and searches will be restricted to only include studies published since Durão and colleagues did their searches in the respective databases [37].

### Data collection and analysis

If any review authors were involved as co‐authors or in any other capacity in any of the studies considered for inclusion, these review authors will not be involved in any of the data collection and analysis processes regarding these studies. In particular, they will not be involved in the study selection process, risk of bias assessment, or synthesis of results.

#### Selection of studies

We will first import all potentially relevant titles and abstracts into Covidence, a software tool for conducting systematic reviews [56]. We will use the software to remove any duplicate results [57]. We will also compare our results to those from Durão and colleagues and exclude any studies that were already screened in the original review [37]. Then, we will screen all titles and abstracts that are left after de‐duplication to determine if they meet our inclusion criteria. Two review authors will carry out this step independently and in duplicate. At the beginning of this stage, we will conduct a calibration exercise with all review authors involved in the screening. If two review authors cannot reach an agreement, the study will be included for full‐text screening.

We will retrieve the full‐text records of any studies that were marked as included at title and abstract screening, and two review authors will independently screen the full texts. Again, we will conduct a calibration exercise with all review authors involved. If the two review authors cannot reach an agreement on inclusion, a third review author will arbitrate.

In addition to screening the full texts identified through our own search and title/abstract screening, we will also screen—independently and in duplicate—all full texts that were included in the title and abstract screening of Durão and colleagues. We will do this to ensure that any studies meeting our eligibility criteria, particularly regarding study designs and outcomes, are not missed due to differences in scope between our review and that of Durão and colleagues [37].

We will record the reasons for exclusion of all ineligible studies and report these according to the PRISMA‐S checklist at this stage. As individual studies, not reports, are our unit of interest, we will identify multiple reports of the same study and link them together before data extraction. If a study fails to report on certain eligibility criteria, but it seems likely that the study will be relevant, we will contact the study authors. If we cannot ascertain the necessary information to determine eligibility after reaching out to authors, we will exclude the study.

We will conduct all screening in Covidence [56].

#### Data extraction and management

Two review authors will independently carry out data extraction. If possible, these review authors will be from complementary disciplines. The current members of the review author team have backgrounds in public health, medicine, nutritional sciences, applied mathematics, economics, and political science. For the studies that were included in the original review by Durão and colleagues, one review author from our team will repeat data extraction [37], with any discrepancies between the data extraction of this review author and the original data extraction resolved by involving a second review author from our team.

We anticipate that some included studies will report more than one outcome measure within a given outcome domain (e.g. total intake of healthy foods and total vegetable intake in the domain 'consumption of healthy foods'). To ensure consistent and transparent selection of outcome measures across studies, we will use a three‐step process for data extraction. First, we will record all outcome measures reported within each outcome domain for each study. This will provide an overview of the outcome measures used across the studies included in the review. Second, we will develop a prioritized list of outcome measures within each outcome domain.

When developing the prioritized list of outcome measures within each outcome domain, we will take the following criteria into consideration.

1. We will give preference to outcome measures included in the core outcome set for early intervention trials to prevent obesity in childhood, 0 to 5 years (COS‐EPOCH) [58]. We anticipate that a core outcome set for school‐based intervention studies on preventing childhood overweight and obesity may become available soon [59]. Should this core outcome set become available before we start data extraction, we will use this newer, more relevant core outcome set.
2. We will give preference to outcome measures that are reported more often in the included studies.
3. We will give preference to broader, more inclusive measures over narrower or more granular ones, as suggested by Hollands and colleagues, i.e. in the above‐mentioned example, we would choose total intake of healthy foods over total vegetable intake [60].
4. We will give preference to outcome measures that we, as review authors, judge to be the most meaningful and relevant for decision‐makers for the respective intervention and outcome domain.

Third, we will extract the reported results for the two outcome measures that are ranked highest within each outcome domain. If a study reports neither of the two highest‐ranked outcome measures within a domain, we will extract the next highest‐ranked measure that is reported.

We acknowledge that this process involves a degree of subjectivity, therefore we will document the rationale for all such decisions transparently.

Outcome selection will be made by the review authors responsible for data extraction and risk of bias assessment and the first review author, with any disagreements resolved through consultation with one of the two last review authors.

We will conduct a data extraction calibration exercise on five studies covering various different intervention types. The list of data extraction items in Supplementary material 2 will be further expanded during data extraction, if necessary. If there are disagreements, a third review author will be consulted to decide. We will keep an updated list of questions with answers and instructions that we will also discuss in regular check‐ins during the data extraction process. If there are multiple reports of one study, we will decide on a case‐by‐case basis whether to extract data for each report separately, or whether one common data extraction is most appropriate. If essential information is missing or not reported in the public data, we will contact the study authors. We will exclude studies for which adequate information for analysis is not provided. We will extract data using Covidence [56].

#### Risk of bias assessment in included studies

The risk of bias assessment investigates and attempts to make transparent the possibility that a study’s results may be skewed due to flaws in its design or execution. As recommended in Chapter 8 of the *Cochrane Handbook for Systematic Reviews of Interventions*, we will conduct risk of bias assessments only for the outcome measures included in the summary of findings tables [61].

##### Randomized studies

We will use the Cochrane RoB 2 tool to evaluate the risk of bias in RCTs according to the guidance provided with the tool and in Chapter 8 of the *Cochrane Handbook for Systematic Reviews of Interventions* [61, 62]. We will focus on the effect of assignment to intervention as the primary effect of interest (intention‐to‐treat analysis), as this is most consistent with the nature of school‐level interventions and the information typically reported in this context. Intention‐to‐treat is an approach to analyzing data that includes all participants as originally assigned, even if they did not fully receive the intervention.

The RoB 2 tool assesses risk of bias based on the following five domains.

1. Bias arising from the randomization process
2. Bias due to deviations from the intended intervention
3. Bias due to missing outcome data
4. Bias in the measurement of outcomes
5. Bias in the selection of the reported results

We will judge each domain as presenting low risk of bias, some concerns, or high risk of bias, based on responses to a series of signaling questions. These domain‐level judgments will contribute to an overall risk of bias rating for each outcome.

For cluster‐randomized trials, we will use the RoB 2 version specifically adapted for this design, which has the added domain 'bias arising from the timing of identification and recruitment of participants,' and follow the guidance provided in Chapter 23 of the *Cochrane Handbook for Systematic Reviews of Interventions* [63].

For cross‐over trials, we will use the RoB 2 version specifically adapted for this design, which has the added domain 'risk of bias arising from period and carryover effects,' and follow the guidance provided in Chapter 23 of the *Cochrane Handbook for Systematic Reviews of Interventions* [63].

For all outcomes of randomized studies, we will designate overall risk of bias as follows.

1. Low risk of bias: all domains are rated low risk.
2. Some concerns: at least one domain raises some concerns, but none are rated high risk.
3. High risk of bias: one or more domains are rated high risk, or multiple domains raise concerns such that confidence in the result may be compromised.

Two review authors will independently assess the risk of bias for each study, with any disagreements resolved through discussion or by consulting a third review author.

We will also consider risk of bias when judging the overall certainty of evidence for each outcome using the GRADE approach. For this purpose, we will classify risk of bias across studies as follows.

1. Low: most of the evidence comes from studies at low risk of bias.
2. Moderate: most evidence comes from studies at low risk or with some concerns.
3. High: high‐risk studies contribute a large proportion of the evidence, potentially affecting interpretation of the findings.

##### Non‐randomized studies

For non‐randomized studies, we will assess the risk of bias using the most recent version of the Risk Of Bias In Non‐Randomized Studies ‐ of Interventions (ROBINS‐I) tool (version 2) [64]. This tool is designed for evaluating specific results from non‐randomized studies that aim to estimate the effect of an intervention on an outcome. It is based on the concept of a 'target trial'—a hypothetical randomized trial that the non‐randomized study attempts to emulate. This approach facilitates assessment of bias in non‐randomized studies by focusing on the estimated causal effect. Risk of bias assessments are conducted per result and guided by structured signaling questions across the following six domains.

1. Bias due to confounding
2. Bias in classification of interventions
3. Bias in selection of participants into the study (or into the analysis)
4. Bias due to missing data
5. Bias arising from measurement of the outcome
6. Bias in selection of the reported result

For each domain, we will judge risk of bias as low, moderate, serious, or critical, with an overall risk of bias judgment derived using the algorithm provided in the tool. As suggested in version 2 of ROBINS‐I, we will shortcut to an overall critical risk of bias assessment, if (1) there is sufficient potential for confounding that an unadjusted result should not be considered further, or (2) the method of measuring the outcome is inappropriate. In such a case, we will not assess the remaining domains.

We will override algorithm‐generated judgments where appropriate, with justification provided, as recommended in the guidance document of the tool.

We will assess the effect of assignment to the intervention (analogous to intention‐to‐treat), as our review focuses on the overall impact of implementing school food environment changes, rather than individual‐level adherence.

Prior to assessments, we will identify and list important confounding factors that studies should ideally control for. Based on our understanding of the topic and initial scoping, and depending on the study design, relevant confounders may include:

1. socioeconomic status of the student population (e.g. eligibility for free or reduced‐price meals);
2. school characteristics (e.g. primary versus secondary level, urban versus rural);
3. pre‐intervention dietary behaviors or weight status;
4. school food policies in place prior to the intervention;
5. seasonal effects;
6. secular trends;
7. implementation‐related variables (e.g. staff engagement, resources available).

We will refine this list based on the characteristics of included studies as needed.

We will follow the guidance provided in Chapter 25 of the *Cochrane Handbook for Systematic Reviews of Interventions* on assessing risk of bias in non‐randomized studies, including studies with quasi‐experimental or other complex designs. This includes how to interpret the ROBINS‐I domains in the context of different study types and how to apply judgment where specific features—such as control for confounding—may vary by design [65].

Two review authors will assess each included study independently, with any disagreements resolved through discussion or by involving a third review author.

Risk of bias assessments will inform the interpretation of findings and the GRADE certainty of evidence ratings.

#### Measures of treatment effect

Given the diversity of outcomes and study designs used in studies about the effects of changing school food environments, we anticipate that included studies will report intervention effects using a range of statistical estimators and descriptors. We will determine which measures of effect are most appropriate for each outcome domain during data extraction, as described in Data extraction and management. We will extract the effect estimates for the chosen outcome measures reported by the included studies. Depending on study design and outcome measure, these may include (standardized) mean differences, change scores, risk ratios, or other measures along with relevant measures of variability.

If both unadjusted and adjusted estimates are available, we will prefer adjusted estimates, provided that they account for relevant confounders. If multiple adjusted models are reported, we will select the one that appears to minimize confounding [66]. If several plausible estimates are available with similar risk of bias, we may extract more than one.

If a study reports multiple measures within the same outcome domain, we will proceed as described above in Data extraction and management. This means that we will prioritize outcome measures based on these criteria: use the outcome measure that (1) is included in a core outcome set; (2) is most consistently used across included studies; (3) is a broader, more inclusive measure; and (4) is most meaningful and relevant to decision‐makers.

Where a study includes more than one intervention or comparator group, we will extract all eligible comparisons. For presentation in summary of findings tables, we will prioritize comparisons between no intervention and the most intensive or comprehensive version of the intervention, or, where applicable, the most stringent versus the least stringent form of implementation.

#### Unit of analysis issues

We anticipate that many of the included studies will use cluster‐level allocation, such as schools or school classes, rather than individual participants. These include cluster‐RCTs, as well as non‐randomized studies where interventions are delivered at the group level, but outcomes may be measured at the individual level.

For cluster‐RCTs, we will account for the clustering in both data extraction and analysis. If studies report effect estimates that appropriately adjust for clustering (e.g. using a multilevel model or generalized estimating equations), we will extract the reported results. If clustering is not accounted for, we will attempt to adjust standard errors using the intracluster correlation coefficient (ICC), following the guidance in Chapter 23 of the *Cochrane Handbook for Systematic Reviews of Interventions* [63]. Where ICC values are not reported, we will attempt to obtain them from study authors or use estimates from similar studies.

For cross‐over designs, we will follow the guidance in Chapter 23 of the *Cochrane Handbook for Systematic Reviews of Interventions* on including variants on randomized trials to ensure that each participant contributes only once to each analysis, thereby avoiding unit of analysis issues [63].

For non‐randomized studies where clustering is part of the design (e.g. prospective controlled studies comparing schools), we will examine whether the analysis appropriately accounts for the clustered structure. If not, we will consider this a potential source of unit of analysis error and assess the impact on risk of bias.

Where studies allocate interventions at the individual level, we do not expect unit of analysis issues. However, for studies that report outcomes at multiple levels (e.g. individual and school‐level outcomes), we will extract results separately for each relevant unit and treat them accordingly in synthesis.

Where a study reports multiple intervention arms or multiple outcome time points, we will ensure that no participants are double‐counted in analyses or summary tables. We will follow the guidance in Chapter 6 of the *Cochrane Handbook for Systematic Reviews of Interventions* on choosing effect measures and computing estimates of effect to avoid unit of analysis errors and to ensure appropriate handling of data from complex study designs [67]. Accordingly, where studies contain multiple arms that compare an intervention with no intervention, we will treat these as if they are separate studies. When the same control group is used as the comparator for different interventions, we will split the shared control group as suggested in Chapter 23 of the *Cochrane Handbook for Systematic Reviews of Interventions* [63].

#### Dealing with missing data

If important information on study characteristics or outcomes is missing and could affect inclusion or interpretation, we will contact the study authors to request additional data. Missing data may include, for example, standard deviations for continuous outcomes, sample sizes, standard errors, or follow‐up times [67]. For trials that have been registered but remain unpublished, we will attempt to contact investigators to obtain relevant data.

Where outcomes are not analyzed according to the intention‐to‐treat principle, we will consider this in our risk of bias assessment under the domain relating to selective outcome reporting. If appropriate, we will explore the impact of including such studies through sensitivity analyses.

#### Reporting bias assessment

Where at least 10 studies are available for meta‐analysis, we will assess the possibility of reporting bias through a funnel plot and conduct statistical tests for funnel plot asymmetry (e.g. Egger’s test) [68]. If fewer than 10 studies are available for meta‐analysis, we will compare published protocols (if available) with published reports to identify any potential selective non‐reporting of results.

#### Synthesis methods

Where at least two studies assess the same intervention type (nutrition standards, marketing restrictions, nudging interventions, pricing policies, and direct food provision) and outcome measure, we will conduct a meta‐analysis using RevMan [69]. When studies use different assessment tools or scales to measure an outcome, we will consider using standardized mean differences (SMD) in data synthesis.

We will undertake separate meta‐analyses for different study designs (e.g. RCTs, interrupted time series) if studies within each intervention type are sufficiently homogeneous. We will pool different types of RCTs (RCTs, cross‐over RCTs, and cluster‐RCTs), provided the last have been adjusted for clustering.

Where studies evaluate multicomponent interventions, we will classify them by the intervention types they include, and group them accordingly for synthesis.

We will use a random‐effects model for meta‐analyses given that we expect considerable diversity in intervention delivery, comparators, setting, and context, following the guidance in Chapter 10 of the *Cochrane Handbook for Systematic Reviews of Interventions* [70]. Specifically, we will use the Restricted Maximum Likelihood (REML) estimator to estimate between‐trial variance. We will use the Hartung‐Knapp‐Sidik‐Jonkman method to calculate a confidence interval for the meta‐analysis effect estimate when there are at least three studies, and the estimate of heterogeneity is greater than zero. In other scenarios (i.e. in pooled analyses of two studies, or where the estimate of heterogeneity is equal to zero), we will use the Wald‐type method [70].

For cross‐over trials, we will follow the guidance in Chapter 23 of the *Cochrane Handbook for Systematic Reviews of Interventions* [63]. Where sufficient data are available to conduct a paired (within‐person) analysis, we will do so, following the procedures recommended in the *Handbook* and as advised by Elbourne and colleagues [71].

If meta‐analysis is not possible due to limited data, substantial heterogeneity, or differences in outcome measurement, we will summarize findings using a narrative approach. We will structure narrative syntheses by intervention type and outcome domain and follow the Synthesis Without Meta‐analysis (SWiM) reporting guideline [72]. If the studies included in narrative syntheses allow for interpretation of the direction of effect, we will use vote counting based on direction of effect, following SWiM guidance. Studies will be grouped by intervention type, and their effects will be categorized as indicating benefit, harm, or no effect. We will summarize results in effect direction plots or harvest plots to support interpretation [72, 73, 74].

Where studies report the same outcome measure at multiple time points, we will use the last time point within the duration of the intervention for data synthesis. In the case that reported outcomes were only observed after the end of the implementation, we will use the first observation taken after the intervention ended. In case only a mean treatment effect over the whole or part of the (post‐)intervention period is reported, we will use this mean for data synthesis.

One review author will prepare the synthesis, which a second review author will check, with any disagreements discussed with review authors involved in data extraction and risk of bias assessment.

#### Investigation of heterogeneity and subgroup analysis

We anticipate that studies will vary in their design, populations, interventions, outcomes as well as outcome measurements. We will explore both clinical and methodological heterogeneity by presenting descriptive tables summarizing key study characteristics. These are listed below.

1. Unit of allocation and level of implementation
2. Intervention subtype (e.g. what type of nudging intervention)
3. Type of school (pre‐, primary, or secondary)
4. Country income category (low‐, middle‐, high‐income country)
5. Population characteristics (e.g. age range, baseline nutritional status, socioeconomic background, gender/sex)
6. Comparators used
7. Use of co‐interventions
8. Outcome types, definitions, and measurement tools
9. Duration of intervention
10. Number of participants whose data were collected and analyzed

For studies examining similar interventions and reporting comparable outcomes, we will assess statistical heterogeneity using both visual and quantitative methods. We will inspect forest plots to examine the degree of overlap in confidence intervals. We will quantify statistical heterogeneity using the I² statistic (to estimate the proportion of variation due to heterogeneity rather than chance), the Chi² test (to test for heterogeneity), and Tau² (to estimate the between‐study variance). We will conduct these analyses using RevMan [69]. We will interpret heterogeneity in line with the guidance in the *Cochrane Handbook for Systematic Reviews of Interventions*. In general, we will consider an I² value greater than 50% as substantial heterogeneity, if either Chi² P value is below 0.10, or Tau² is greater than zero. However, decisions on synthesis will also consider consistency in the direction of effects, number of studies, and relevance of comparisons.

Where meaningful differences in study characteristics are observed, we will explore potential explanations through subgroup analyses, if data permit. Any such analyses will be considered strictly exploratory.

If data allow (if there are at least 10 studies for any given intervention domain and outcome domain combination that reported disaggregated data), we will conduct subgroup analyses by either performing a separate meta‐analysis, or creating separate harvest or effect direction plots to examine the differential effects of the following subgroups.

1. Schooling level (pre‐, primary, secondary)
2. Income level of the country where the study was conducted (high‐income, upper‐middle‐income, lower‐middle‐income, and low‐income countries), according to the World Bank definition [75]
3. If feasible, single interventions versus multicomponent interventions (including intervention components that are not one of the five intervention types that we included in this review)
4. Relevant factors from the PROGRESS‐Plus framework, as described below [76, 77]

For prespecified subgroup analyses, we will conduct random‐effects meta‐analyses within each subgroup in RevMan, using the same model as in the main analyses [69]. To assess whether intervention effects differ between subgroups, we will use the 'test for subgroup differences' in RevMan, which performs a Chi² test and provides an I² statistic for subgroup differences, following the guidance in Chapter 10 of the *Cochrane Handbook for Systematic Reviews of Interventions* [69, 70].

##### Equity‐related assessment

We will seek to assess whether the effects of school food environment interventions differ across population groups, in line with the guidance in Chapter 16 of the *Cochrane Handbook for Systematic Reviews of Interventions* [78]. We aim to understand whether these interventions contribute to reducing or widening health and nutrition‐related inequities.

To do this, we will consider the PROGRESS‐Plus framework, which includes the following equity‐relevant factors.

1. Place of residence (e.g. urban versus rural settings)
2. Race/ethnicity/cultural identity/language
3. Occupation (e.g. parental employment or school‐level characteristics)
4. Gender/sex
5. Religion
6. Education (e.g. parental education or school‐level educational indicators)
7. Socioeconomic status (e.g. income level, eligibility for free or reduced‐price meals)
8. Social capital
9. Plus: other personal or contextual characteristics such as disability or migrant status

Based on prior reviews and policy relevance, we will prioritize the following three equity dimensions for closer examination [77].

1. Socioeconomic status, including both direct indicators and baseline nutritional status as a proxy
2. Gender/sex (as reported in the studies)
3. Rurality, as well as neighborhood disadvantage within urban settings (e.g. whether the school is located in a lower‐income or underserved area)

During data extraction, we will record whether studies report subgroup analyses or disaggregated outcomes by any of these three dimensions. If such data are available, we will extract and report them in the results. Where appropriate, we may conduct subgroup analyses on these equity factors, as described above.

We will also assess whether equity considerations were embedded in the design or targeting of the interventions, for example whether programs were specifically implemented in schools serving disadvantaged populations.

#### Sensitivity analysis

To assess the robustness of our findings, we will conduct sensitivity analyses where feasible. These will focus on testing the impact of potential sources of bias. Specifically, we will exclude studies assessed as having high risk of bias (for randomized trials) or serious or critical risk of bias (for non‐randomized studies). Where sufficient data are available, we will conduct separate meta‐analyses of the remaining studies. If meta‐analysis is not feasible, we will examine effect direction plots or harvest plots.

#### Certainty of the evidence assessment

We will assess the certainty of the evidence for all intervention and outcome combinations using the GRADE approach. This framework helps review authors judge how confident we can be that the estimated effects reflect the true impact of the intervention.

In line with GRADE guidance on choosing targets of GRADE certainty of evidence ratings [79], and following the approach used in other Cochrane reviews on public health measures [80, 81, 82], we will consider any difference from the null as the minimally important threshold when assessing the certainty of evidence. Our GRADE assessments will therefore focus on the existence and direction of effect, rather than on the magnitude of effect sizes. However, we will transparently document any cases where effect sizes appear very small and may raise questions about practical or clinical relevance.

We will create separate summary of findings tables for each intervention type, that is the following five comparisons: (1) nutrition standards or rules determining the quality of foods served or sold in and around schools versus no intervention; (2) marketing restrictions on unhealthy foods to children in and around schools versus no intervention; (3) nudging interventions promoting healthy diets versus no intervention; (4) pricing interventions that alter the relative costs of healthy and less healthy foods versus no intervention; and (5) direct food provision to students in schools versus no intervention.

Within each summary of findings table, we will include the five critical outcome domains and outcome measures selected based on the predefined ranking approach described in Data extraction and management. Within each critical outcome domain, we will report at most two outcome measures, and we will limit the total number of outcome measures reported in each summary of findings table to a maximum of seven. If there are no data available from included studies for an outcome measure selected for the summary of findings tables, we will document and report this in the final review. For each outcome measure, we will use the time point of interest as described in Outcome measures. Where different methods of measurement or scales were used, we may present results using SMDs. We will report outcome measures that were extracted but not chosen as one of the seven outcome measures for a given summary of findings table in the supplementary material of the final review.

In GRADE, the certainty of evidence is initially set at high certainty for both evidence from RCTs assessed with RoB 2 and non‐randomized studies of interventions assessed with ROBINS‐I, acknowledging that the latter compares results against a hypothetical well‐conducted target trial. In line with GRADE guidance, this high starting point for non‐randomized studies reflects an intention to treat all studies on a common risk of bias scale, rather than assuming lower certainty based solely on study design [83]. In practice, however, studies rated as having serious or critical risk of bias across multiple domains in the ROBINS‐I assessment will typically lead to downgrading by up to three levels. Specifically, certainty may be downgraded based on the following factors.

1. Risk of bias: limitations in study methods that may affect the validity of findings (as assessed with RoB 2 or ROBINS‐I)
2. Inconsistency: unexplained variability in direction of effects across studies
3. Indirectness: differences between the study characteristics and the population, intervention, comparator, or outcome of interest
4. Imprecision: wide confidence intervals, small sample sizes, or few events
5. Publication bias: evidence or suspicion that studies are missing due to non‐reporting of negative or null findings

All decisions to downgrade the certainty of the evidence will be made transparently and documented in the summary of findings tables, with explanations provided for each judgment.

In addition to the above factors, we will consider reducing the extent of downgrading the certainty of evidence in cases where a credible dose‐response gradient (DRG) is observed, in accordance with the latest GRADE guidance [84]. A DRG will be considered credible when established through a robust analytical approach. In the absence of suitable data for regression‐based approaches, we will examine whether effect estimates across at least three ordered subgroups (e.g. low, moderate, high intensity) display a consistent and monotonic trend. For a DRG based on subgroup data to be deemed credible, we will require that: (1) exposure levels are ordinal and defined by the review authors before analysis; (2) effect estimates are available for each level from at least two studies, ideally more; (3) the observed trend is biologically or theoretically plausible; and (4) there is no serious concern about residual confounding, ecological bias, or inconsistency in the DRG across studies. If these conditions are met, we will consider reducing the degree of downgrading by one level, in accordance with GRADE guidance [84].

In rare cases, we may reduce the extent of downgrading the certainty of evidence due to a large effect, in line with GRADE guidance in the context of ROBINS‐I assessments [83]. We will only consider this when effect estimates are large (i.e. relative effect > 2.0 or < 0.5) and accompanied by narrow confidence intervals, and where the magnitude of the effect renders confounding or other biases an implausible alternative explanation [83, 85]. In such cases, large effects may serve as a reason to reduce the extent of downgrading by one level.

Both upgrading criteria will be applied conservatively and only when the strength and consistency of the evidence clearly support this interpretation. We will not reduce downgrading if there is serious concern about residual confounding.

Any decisions will be transparently documented with explicit rationale.

Ratings will be agreed upon by at least two review authors, with any disagreements resolved through discussion or arbitration by a third review author if necessary.

### Consumer involvement

We aim to involve relevant stakeholders throughout the review process to ensure the findings are meaningful and applicable to end users. For this review, we define 'consumers' broadly to include individuals who may be affected by or involved in school food environment interventions and policies, such as students, school leadership (e.g. headteachers), parents, and teachers. We also consider as stakeholders organizations that are involved in school food environment policy or implementation, including government bodies, NGOs, and multilateral or international agencies working at national or global levels.

We have established a Review Advisory Group to provide input at key stages. Prior to finalizing the protocol, members of the group were consulted to review and rank outcome domains and outcome measures, and to provide feedback on the descriptions of intervention types. Their contributions informed the final selection of outcomes and refined the intervention descriptions.

During the review process, we will continue to consult the Review Advisory Group on substantive interpretive issues, such as the relevance of findings for decision‐makers and the interpretation of complex or inconsistent evidence.

We will document the nature, timing, and purpose of any involvement from the Review Advisory Group and other stakeholders in a supplementary file to the final review.

## Supporting Information

Supplementary materials are available with the online version of this article: 10.1002/14651858.CD015882.

Supplementary materials are published alongside the article and contain additional data and information that support or enhance the article. Supplementary materials may not be subject to the same editorial scrutiny as the content of the article and Cochrane has not copyedited, typeset or proofread these materials. The material in these sections has been supplied by the author(s) for publication under a Licence for Publication and the author(s) are solely responsible for the material. Cochrane accordingly gives no representations or warranties of any kind in relation to, and accepts no liability for any reliance on or use of, such material.

**Supplementary material 1** Search strategies

**Supplementary material 2** Data extraction items

## Additional information

### Registration and protocol

Cochrane approved the proposal for this review in March 2023.

### Data, code and other materials

Data sharing not applicable to this article as it is a protocol, so no datasets were generated or analysed.
