# Supplementary material for: School food environment interventions for health and sustainability
Source: Cochrane Database Syst Rev. 2026 Mar 17;2026(3):CD015882. doi: 10.1002/14651858.CD015882 (PMC12994130; doi:10.1002/14651858.CD015882)
Supplement: Supplementary file 1 — Supplementary material 1 Search strategies [file CD015882-SUP-01-searchStrategy.html]

Search strategies


# Supplementary material 1 to: School food environment interventions for health and sustainability

Leibinger A, Holliday N, Klinger C, Tan X, Busert-Sebela L, Schwingshackl L, Rehfuess E, Durao S, von Philipsborn P
  
https://doi.org/10.1002/14651858.CD015882

The material in this section has been supplied by the author(s) for publication under a Licence for Publication and the author(s) are solely responsible for the material. Cochrane has peer reviewed this material in accordance with its editorial policies, but Cochrane has not copyedited, formatted or proofread. Cochrane accordingly gives no representations or warranties of any kind in relation to, and accepts no liability for any reliance on or use of, such material.

Back to top

# Search strategies

This is the original search strategy adapted for use in Ovid Medline:

1. exp Food Services/ or exp Food Preferences/ or exp Food/st or exp Health Promotion/ or exp School Health Services/ or exp Nutrition Policy/ or exp Food Legislation/ or exp Dietary Fats/st or exp Feeding Behavior/

2. (school-based intervention or school-based interventions or food service intervention or food service interventions or foodservice intervention or foodservice interventions or cafeteria-based intervention or cafeteria-based interventions or nudging or lunch program or lunch programme or lunch programs or lunch programmes or meal program or meal programme or meal programs or meal programmes or breakfast program or breakfast programme or breakfast programs or breakfast programmes or nutrition program or nutrition programme or nutrition programs or nutrition programmes or food program or food programme or food programs or food programmes or food service program or food service programme or food service programs or food service programmes or foodservice program or foodservice programme or foodservice programs or foodservice programmes or school-based program or school-based programme or school-based programs or school-based programmes or nutrition policy or nutrition policies or school food policy or school food policies or food service policy or food service policies).ti,ab.

3. ((food or foodservice or nutrition\* or meal or canteen or cafeteria\* or lunch or breakfast or school-based) AND (legislation or legislations or law or laws or ban or bans or restrict\* or mandate\* or guideline or guidelines)).ti,ab.

4. ((food or meal or lunch or breakfast) AND (provision or distribution or selection or promotion or promotions or prices or pricing or procurement or product placement\*)).ti,ab.

5. 1 or 2 or 3 or 4

6. exp Diet/ or exp Beverages/ or exp Sodium, Dietary/ or exp Energy Intake/ or exp Dietary Fats/ or (diets or food or foods or nutrition or beverage or beverages or soda or fruit or fruits or vegetable or vegetables or drinking water or salt or sugar or breakfast or meal or meals or lunch or lunches or fat or fats).ti,ab.

7. exp Schools/ or exp Child Care/ or (school or schools or schoolchildren or child care or child day care or child daycare or childcare or day care center\* or day care centre\* or daycare center\* or daycare centre\* or creche\* or aftercare or after care or preschool\* or after-school care or day nursery or day nurseries or pre-primary or kindergarten\* or kindergarden\*).ti,ab.

8. exp randomized controlled trial/ or controlled clinical trial.pt. or randomized.ab. or placebo.ab. or drug therapy.fs. or randomly.ab. or trial.ab. or groups.ab.

9. exp animals/ not humans.sh.

10. 8 not 9

11. comparative study.pt. or exp Control Groups/ or exp Follow-Up Studies/ or exp Prospective Studies/ or exp “Evaluation Studies as Topic”/ or exp Interrupted Time Series Analysis/ or exp Controlled Before-After Studies/ or (controlled or CBA stud\* or pre test or pretest or post test or posttest or pre intervention or post intervention or before or after or groups or follow-up stud\* or follow-up assessment or evaluat\* or quasi experiment\* or quasiexperiment\* or prospective stud\* or ITS stud\* or time series or time point\*)ti,ab.

12. 10 or 11

9. 5 and 6 and 7 and 12
