# Supplementary material for: School food environment interventions for health and sustainability
Source: Cochrane Database Syst Rev. 2026 Mar 17;2026(3):CD015882. doi: 10.1002/14651858.CD015882 (PMC12994130; doi:10.1002/14651858.CD015882)
Supplement: Supplementary file 2 — Supplementary material 2 Data extraction items [file CD015882-SUP-02-other.html]

Data extraction items


# Supplementary material 2 to: School food environment interventions for health and sustainability

Leibinger A, Holliday N, Klinger C, Tan X, Busert-Sebela L, Schwingshackl L, Rehfuess E, Durao S, von Philipsborn P
  
https://doi.org/10.1002/14651858.CD015882

The material in this section has been supplied by the author(s) for publication under a Licence for Publication and the author(s) are solely responsible for the material. Cochrane has reviewed this material, but Cochrane has not copyedited, formatted or proofread. Cochrane accordingly gives no representations or warranties of any kind in relation to, and accepts no liability for any reliance on or use of, such material.

Back to top

# Data extraction items

**Basic Information on Data Extraction**

- Review author initials
- Date of data extraction
- Study ID

**General Information on Study**

- Study/report title
- Corresponding author name and email
- Year of publication
- Language of publication
- Declaration of Interest reported
- Funding source
- Funding source category (e.g. government, industry)
- Study or programme name or acronym
- Type of record
- Study aim or objective
- Conflicts of interest of all authors involved, as stated in the included reports

**Intervention and comparison**

- Setting and context
  - Country
  - Focus on disadvantaged areas within the country/city?
  - Schooling level (e.g., preschool, primary, secondary)
  - In-school, after school, or around school
  - Rurality
  - Implementation level (e.g. class, school, nation-wide)
- Intervention characteristics for intervention and control separately
  - Intervention type
  - Intervention subcategory if applicable
  - Intervention description as provided by study authors
  - Duration of the intervention
  - Co-interventions
  - Staffing resources needed
  - Financial resources needed
- Comparison (no intervention, minimal intervention, or alternative intervention)

**Study design and data collection methods**

- Study design
- Study grouping (e.g. crossover, parallel, cluster)
- Unit of allocation/exposure: states, districts, schools, classes, or individuals
- Study length (time period between first and last data assessment)
- Number and timepoints when outcomes were measured
- Consideration of equity aspects

**Participants**

- Baseline characteristics for intervention and control separately, and overall:
  - Age
  - Sex/gender (as reported in the studies)
  - Socioeconomic status (of parents)
  - Baseline nutritional status
- Description of subgroups measured and reported
- Total number completed and analyzed, and per relevant group

**Outcomes**

- For each of the following outcome domains, if reported by relevant subgroups and totals: Effect estimator, measures of uncertainty (e.g., standard error, confidence interval), unit of analysis, measurement tool/definition, and method of aggregation:
  - Consumption of healthy foods (e.g., proportion/quantity/frequency of consumption of healthy foods or meals in general, or specific foods such as fruits and vegetables, nuts, legumes, whole grains);
  - Consumption of discretionary foods (e.g., proportion/quantity/frequency of discretionary foods in general, or of specific foods such as sugar-sweetened beverages or energy-dense snacks and sweets);
  - Overweight and obesity (i.e., prevalence or incidence of overweight and/or obesity, changes in BMI);
  - Undernutrition (i.e., prevalence or incidence of underweight, stunting, wasting, or micronutrient deficiencies);
  - Any adverse health effects (e.g. eating disorders, body image disorders);
  - Sustainability measures (amount of food waste and greenhouse gas emissions from foods);
  - Educational outcomes (e.g., school absenteeism and educational attainment).

Besides these items, we will extract all data necessary for the risk of bias assessment using RoB 2 and ROBINS-I, as outlined in the section on Risk of bias assessment in included studies.
